# Utilizing the TrueBeam Advanced Imaging Package to monitor intrafraction motion with periodic kV imaging and automatic marker detection during VMAT prostate treatments

**DOI:** 10.1002/acm2.12822

**Published:** 2020-01-24

**Authors:** Mark C. Korpics, Michelle Rokni, Michael Degnan, Bulent Aydogan, Stanley L. Liauw, Gage Redler

**Affiliations:** ^1^ Department of Radiation and Cellular Oncology University of Chicago Medical Center Chicago IL USA

**Keywords:** fiducial tracking, IGRT, intrafraction motion management, marker tracking, prostate

## Abstract

**Background:**

Fiducial markers are frequently used before treatment for image‐guided patient setup in radiation therapy (RT), but can also be used during treatment for image‐guided intrafraction motion detection. This report describes our implementation of automatic marker detection with periodic kV imaging (TrueBeam v2.5) to monitor and correct intrafraction motion during prostate RT.

**Methods:**

We evaluated the reproducibility and accuracy of software fiducial detection using a phantom with 3 implanted fiducial markers. Clinical implementation for patients with intraprostatic fiducials receiving volumetric modulated arc therapy (VMAT) utilized periodic on‐board kV imaging with 10 s intervals during treatment delivery. For each image, the software automatically identified fiducial locations and determined whether their distance relative to planned locations were within a 3 mm tolerance. Motion was corrected if either ≥2 fiducials in a single image or ≥1 fiducial in sequential images were out of tolerance.

**Results:**

Phantom studies demonstrated poorer performance of linear fiducials compared to collapsible fiducials, and wide variability to accurately detect fiducials across eight software settings. For any given setting, results were relatively reproducible and precise to ~0.5 mm. Across 17 patients treated with a median of 20 fractions, the software recommended a shift in 44% of fractions, and a shift was actually implemented after visual confirmation of movement greater than the 3 mm threshold in 20% of fractions. Adjustment of our approach led to improved accuracy for the latter (n = 7) patient subset. On average, table repositioning added 3.0 ± 0.3 min to patient time on table. Periodic kV imaging increased skin dose by an estimated 1 cGy per treatment arc.

**Conclusions:**

Periodic kV imaging with automatic detection of motion during VMAT prostate treatments is commercially available, and can be successfully implemented to mitigate effects of intrafraction motion with careful attention to software settings.

**Abbreviations**
IGRT: imaged‐guided radiotherapy
OBI: on‐board‐imager
RT: radiation therapy
VMAT: volumetric modulated arc therapy

## 1 Introduction

Prostate setup variation over a course of radiotherapy (RT) can compromise treatment, especially for highly conformal treatments using volumetric modulated arc therapy (VMAT), in which steep dose gradients are devised to limit radiation to adjacent normal tissues. Image‐guided radiotherapy (IGRT) using implanted fiducial markers can reduce setup error, and potentially improve treatment efficacy and decrease treatment related morbidity.^1^, ^2^, ^3^, ^4^, ^5^, ^6^ Most IGRT approaches address interfraction motion at the start of each treatment delivery, but do not account for intrafraction motion, which can occur due to physiologic bladder or rectal motion, or patient repositioning during treatment. Electromagnetic tracking and targeting,^7^, ^8^ along with the Calypso 4D Localization System (Calypso Medical Technologies, Seattle, WA), offers one solution, but a more readily available method for those without such specialized equipment would be valuable.

The Varian Truebeam v2.5 (Advanced IGRT & Motion Package, Varian Medical Systems, Palo Alto, CA) enables planar kV images to be acquired during treatment delivery using the orthogonal on‐board‐imager (OBI). The software uses a proprietary search algorithm to find the location of the fiducials in each triggered image and determines if they are within a predetermined tolerance from the expected location. In this report, we present our experience implementing this feature into our clinical workflow. We initiated a process of evaluating this software for intra‐fraction monitoring in VMAT to the prostate, including work using a phantom, a pilot experience of ten patients undergoing therapy, and analysis including seven subsequent patients with a refined approach (i.e., 17 total), under approval from an institutional review board.

**Figure 1:**
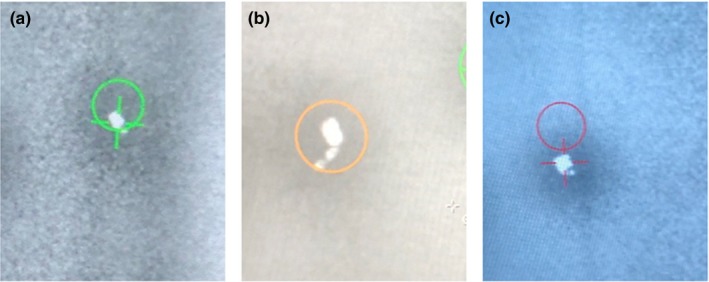
Colored circles represent the search region for a given tolerance and crosshairs represent the found fiducial location. Green signifies that the fiducial is within tolerance (a), Yellow signifies that the fiducial was not found (b), and Red signifies that the fiducial is out of tolerance (c).

The software package evaluated allows the user to define the frequency of triggered kVs based on various criteria: breathing motion of the patient, elapsed time, number of MU delivered, or gantry angle. The user further defines the predetermined tolerance of relating the position of the identified marker to the expected position (the center of mass of the high‐resolution manual contour of the fiducial). The search region is depicted as a colored circle and the identified marker location as a crosshair (Fig. 1). The circle and crosshair are shown in three colors: green, if the fiducial is within tolerance; yellow, if the software could not find the fiducial; and red, if the fiducial is out of tolerance. This visualization allows the user to qualitatively verify any fiducial shifts during treatment.

## 2 Methods

**Figure 2:**
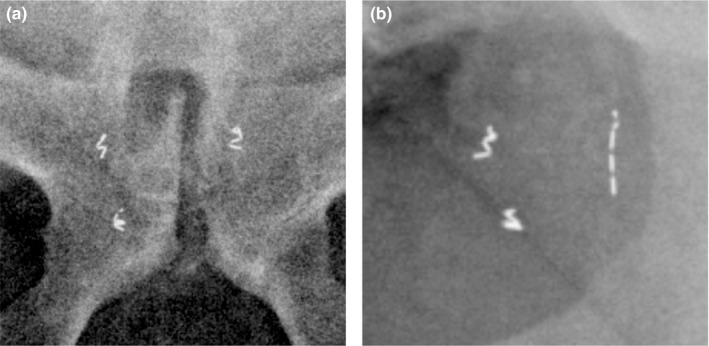
Example of a patient with three crumpled fiducials (a), and a patient with two crumpled fiducials and one linear fiducial (b).

**Figure 3:**
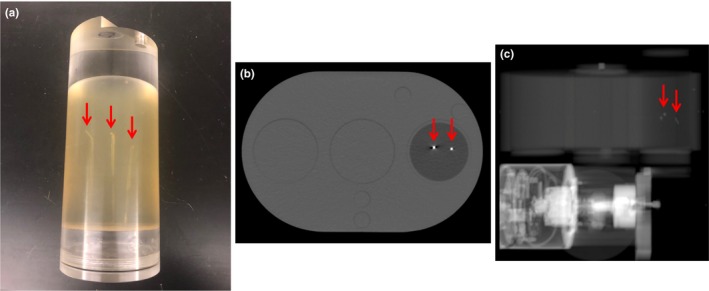
Phantom constructed with gelatin and three collapsible gold fiducial markers: photograph of cylinder (a), axial CT image (b), and kV image (c). Arrows indicate the fiducial locations.

A phantom was created to mimic patient composition and geometry with three 0.28 × 10 mm gold collapsible fiducial markers implanted using a 22G, 20 cm needle (Gold Anchor, Naslund Medical, Sweden). These fiducials can be placed in either a linear or crumpled orientation (Fig. 2). The phantom consisted of a container of gelatin containing two crumpled and one linear fiducial marker (Fig. 3), inserted into a larger, acrylic phantom (QUASAR™ Multi‐Purpose Body Phantom, Modus QA, Canada) to evaluate specific aspects of automatic fiducial detection and intrafraction motion monitoring. Specific factors evaluated included effect of fiducial type, reproducibility, and accuracy. A single treatment arc with a static phantom was used for all phantom analyses. This software also offers multiple settings for marker detection with the following descriptions: “CalypsoTransponder,” “Clip_1_5x4_0,” “EmbolizationCoil_3_0x3_0,” “EmbolizationCoil_4_0x4_0,” “GoldSeed_1_0x3_0,” “GoldSeed_1_3x5_0,” “GoldSeed_1_8x3_6,” and “GoldSeed_2_5x5_0.” For example, “Clip_1_5x4_0” is a software setting that would be expected to perform well when using surgical clips as fiducial markers. The fiducial‐type setting was evaluated by delivering a single treatment arc with kV images acquired every 3 s (20 images/arc or equivalently 20 images/fiducial‐type setting) for each of the eight available fiducial type settings using a 3 mm radius tolerance.

Three fiducial‐type settings (“Clip_1_5x4_0,” “GoldSeed_1_3x5_0” and “GoldSeed_1_8x3_6”) were chosen for superiority in automatic detection for additional phantom studies to evaluate accuracy and reproducibility. To assess accuracy, automatic marker detection was utilized with the phantom correctly aligned (shift = 0.0 mm) as well as with offsets (~0.5 mm increments) purposefully introduced via phantom positioning shifts in the superior‐inferior direction.. With the phantom in these different positions, a single treatment arc was used with triggered kV images acquired every 20 degrees. This enabled testing of the capability to identify when the fiducials moved outside of the 3 mm tolerance. The reported positional accuracy for this treatment couch (BrainLab AG, Feldkirchen, Germany) is 0.07 ± 0.22 mm.^9^ Reproducibility was assessed by delivering the same treatment arc three times in a row while maintaining the same phantom position and observing discrepancies in repeated measurements.

Clinical performance of the software was evaluated on a total of 17 prostate cancer patients with intra‐prostatic collapsible fiducials (an initial cohort of ten followed by an additional cohort of seven, for which the approach was refined based on experience with initial cohort). The University of Chicago Institutional Review Board (IRB) approved this study (IRB14‐934A). Fourteen patients had three crumpled fiducial markers (group A), while three patients had at least one linear fiducial marker (group B). An 18^th^ patient with cylindrical fiducial markers placed at an outside institution was excluded, as it was found that the selected fiducial marker settings performed poorly. All patients had Gold Anchor fiducials placed transrectally prior to RT. RT was delivered with a VMAT technique using 2‐3 arcs and 6 MV photons. Triggered kV images were acquired every 10 s using the OBI, with fiducial‐type detection settings of “GoldSeed_1_3x5_0” (n = 7) or “Clip_1_5x4_0” (n = 10). Intrafraction motion correction with a couch shift was implemented when two fiducial markers in a single kV image were outside of set 3 mm tolerance, or when the same fiducial marker in two sequential images was outside of tolerance. Intrafraction correction required a treatment pause, acquisition of new orthogonal kV images utilizing on demand kV‐kV pair imaging (i.e. the two orthogonal kV images are acquired at the gantry position where the treatment stopped and not necessarily at 0 and 90 degrees), registration of the kV image to CT simulation image, alignment of fiducial markers, and manual couch shift. During treatment delivery, radiation therapists visually verified the automatic fiducial marker detection before treatment was stopped, as the automated algorithm did not have perfect sensitivity or specificity. When the software suggested a shift based on predefined criteria, the therapists would qualitatively confirm or reject whether the shift was necessary. If the software was unable to identify the fiducials, then treatment continued unless the therapists noted the fiducial to be outside of tolerance.

Planned fiducial locations, for both phantom experiments and clinical implementation, were determined using the planning CT (120 kVp, 93 mA). The fiducials were manually contoured using the CT and the marker location was set as the software identified centroid.

## 3 Results

**Figure 4:**
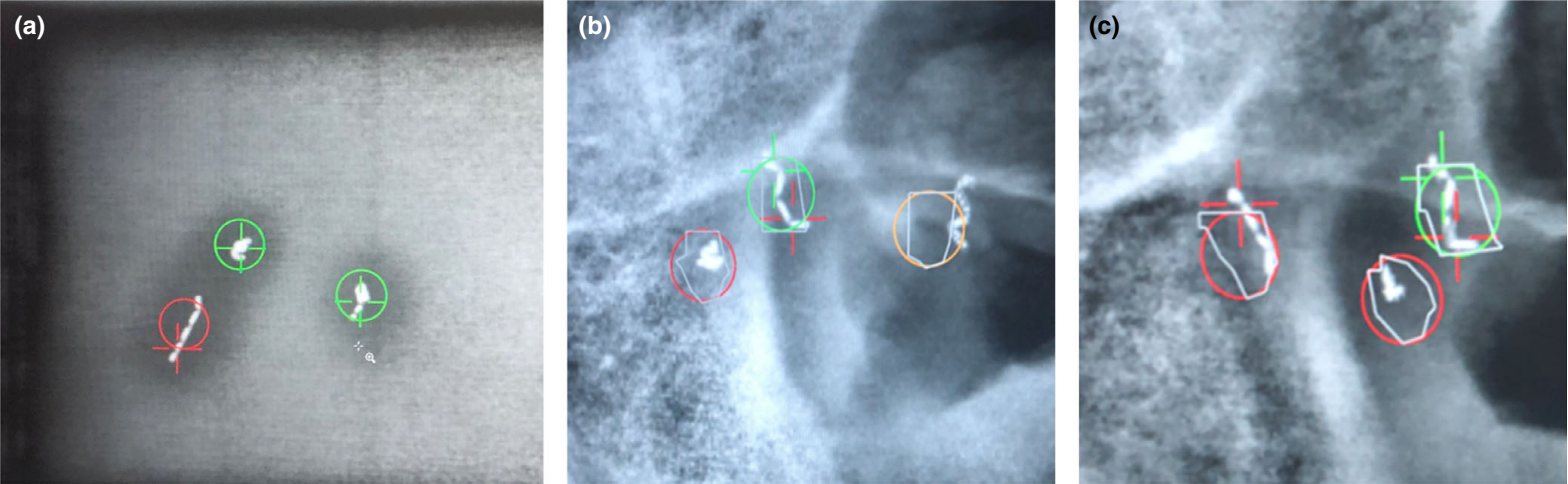
Linear fiducial markers were often inaccurately identified as compared to crumpled fiducial markers as shown in phantom studies (a) and patient studies (b) & (c).

**Figure 5:**
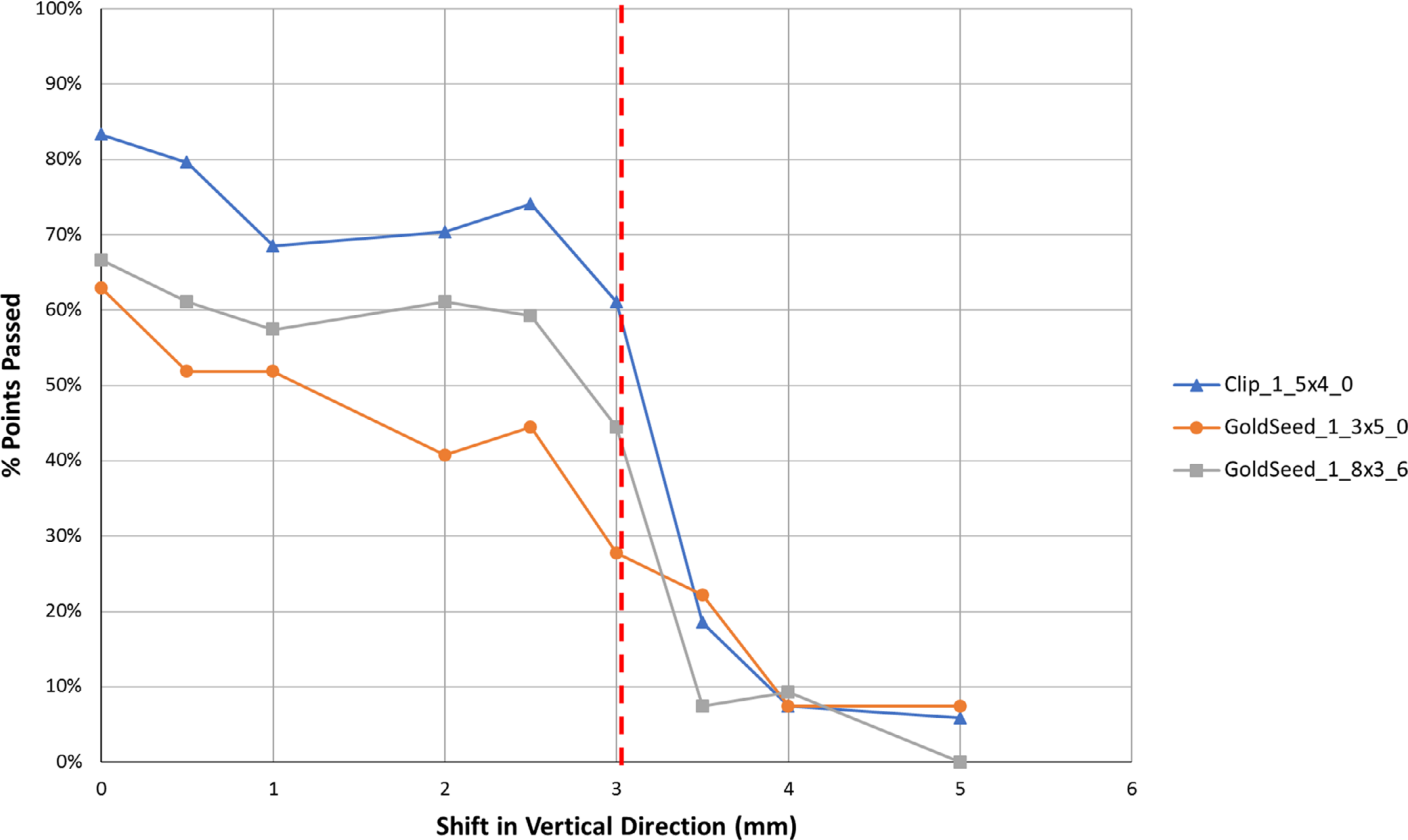
Percentage of the kV images, in which the fiducial marker was identified and found to be within tolerance versus the table shift magnitude. The pre‐defined tolerance of 3 mm is depicted as a vertical red dotted line.

The phantom analysis revealed that linear fiducials were often inaccurately identified or not found as compared to crumpled fiducials (Fig. 4). Figure 4(a) depicts an instance in which the software incorrectly identified the center of mass of the linear fiducial, and subsequently labeled the marker as out of tolerance, despite the fact that it appears within the circle. Figure 4(b) depicts an instance in which the linear fiducial was not found (i.e. right‐most yellow circle). Figures 4(b) and 4(c) depict instances in which the software identified the inferior aspect of the linear fiducials as the crumpled fiducials, which caused the software to label the adjacent crumpled fiducials as out of tolerance, despite the fact that they appear to be within tolerance (i.e. within the red circle). When assessing the effect of using different fiducial‐type settings, “Clip_1_5x4_0” performed the best as depicted in Table 1. Given this finding, the acquisition of clinical data was eventually changed from the “GoldSeed_1_3x5_0” setting to the “Clip_1_5x4_0” setting. Using the three fiducial‐type settings that performed the best (“Clip_1_5x4_0,” “GoldSeed_1_3x5_0” and “GoldSeed_1_8x3_6”), fiducial marker positions were found to be reproducible with 0.5 mm of precision. Using these three settings, accuracy was assessed by shifting the table in increments of 0.5 mm from the initial position and assessing the number of kV images in which the fiducial markers were correctly identified within 3 mm of tolerance (Fig. 5).

**Table 1:** Effect of fiducial‐type software setting on the accuracy of fiducial marker identification within the phantom.

Fiducial marker type	% Passed	% Not found	% Failed
CalypsoTransponder	77	8	15
Clip_1_5x4_0	88	5	7
EmbolizationCoil_3_0x3_0	42	53	5
EmbolizationCoil_4_0x4_0	39	34	27
GoldSeed_1_0x3_0	60	33	7
GoldSeed_1_3x5_0	60	17	23
GoldSeed_1_8x3_6	70	30	0
GoldSeed_2_5x5_0	63	13	23

Columns show percentage of kV images, in which the fiducial was correctly identified and within tolerance (passed), could not be identified (not found) and was identified but was out of tolerance (failed). Standard deviation was 3% overall.

**Figure 6:**
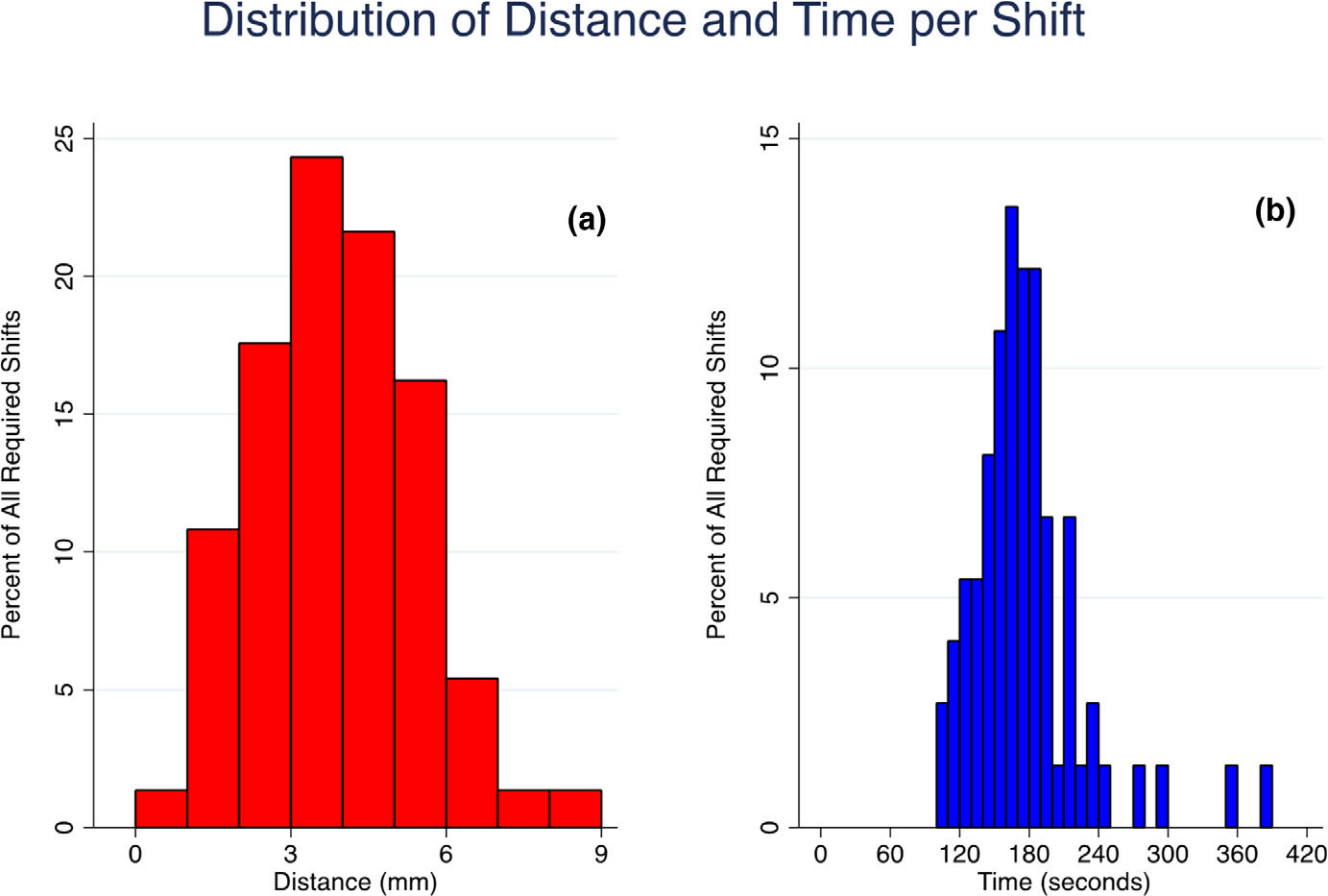
Histogram showing the distribution of the magnitude of the distance per shift (a) and the time required per shift (b). Time 0 is defined as the time that pre‐treatment orthogonal pair kV has been completed.

A summary of the software's performance for each patient is shown in Table 2. Out of 351 total fractions, the software suggested shifts in 156 fractions (421 instances). After visual verification, shifts were made only in 69 out of 351 fractions (74 shifts). Of all 421 instances in which the software suggested a shift, a shift was made 74 times (18%, 39% for group A vs. 4% for group B). Sixteen of 17 (94%) patients had at least one shift over their treatment course; on a patient level, an intrafraction shift was implemented in 17% of all fractions (range, 0‐53%). Of the 74 total shifts, 24% (8 patients, 5% of total fractions) were ≥5 mm, 46% (15 patients, 10% of total fractions) were 3‐5 mm, and 30% were ≤3 mm (10 patients, 6% of total fractions), respectively. Shifts of magnitude 3 and 5 mm were chosen to represent movement beyond two common PTV margins (see Fig. 6). The average duration of treatment was 5.5 ± 0.5 min to deliver a median of two VMAT arcs, and each shift made from the triggered kVs required an average interruption of 3.0 ± 0.3 min. The average shift magnitude was 3.5 ± 1.1 mm. There was no significant angular dependence for the auto‐detection of fiducial markers. Specifically, there was no observed degradation in auto‐detection when the imaging arms were near 90 and 270 degrees, which correspond to images subject to obfuscation of prostatic fiducials by the highly attenuating hips of each patient. Regarding patient size, the average body mass index of the entire patient cohort was 29 ± 5 kg/m^2^. Patient size was not found to be a factor in the identification of markers. For the excluded patient with cylindrical fiducials, using the same settings as in the other 17 patients, the software found that in 45% of kV images the fiducial was correctly identified and within tolerance, representing a very low rate of accurate detection.

**Table 2:** Percentage of kV images, in which the fiducial was correctly identified and within tolerance (passed), could not be identified (not found) and was identified but was out of tolerance (failed).

Patient	% Passed	% Not found	% Failed	Number of fiducial markers (crumpled/linear)	% Instances requiring correction	% Fractions needing shift
Patient 1^A^	68	13	6	3/0	100	50
Patient 2^A^	80	25	6	3/0	33	15
Patient 3^B^	58	8	34	0/2	1	5
Patient 4^B^	65	10	25	2/1	7	25
Patient 5^A^	53	42	6	3/0	39	26
Patient 6^A^	95	2	3	3/0	50	15
Patient 7^B^	56	30	14	2/1	5	10
Patient 8^A^	83	5	12	3/0	45	53
Patient 9^A^	89	5	6	3/0	30	17
Patient 10^A^	80	14	6	3/0	50	17
Patient 11^A^	75	19	6	3/0	40	32
Patient 12^A^	90	7	4	3/0	33	15
Patient 13^A^	93	2	5	3/0	31	21
Patient 14^A^	100	0	0	3/0	N/A	0
Patient 15^A^	85	10	4	3/0	17	10
Patient 16^A^	91	0	8	3/0	25	20
Patient 17^A^	91	4	5	3/0	80	10

When two fiducials in a single kV image were out of tolerance, or a single fiducial in sequential kV images was out of tolerance, a shift was required (fractions needing shift). Of note, five fractions actually required two shifts each. The percentage of instances requiring correction refers to instances in which a shift was required out of all instances, in which a shift was suggested. Superscripts refer to the corresponding patient group (A, 3 crumpled fiducial markers; B, at least one linear fiducial marker).

## 4 Discussion

As compared to Calypso, which can continuously monitor intrafraction motion, this approach represents a cheaper, more readily available, and more efficient method of correcting intrafraction motion.^10^ Our findings are concordant with prior studies of Calypso in that there are different motion profiles during each treatment session with no obvious temporal trend to large movements.^11^ Longer treatment times are associated with more frequently observed small movements, but this is less relevant with the shorter treatment times of VMAT. Since Calypso uses an electromagnetic tracking and targeting system, there is no added ionizing radiation dose as compared to this approach. However, assuming little overlap in the skin for each individual kV image, only ~1 cGy will be deposited uniformly to the skin per treatment arc, resulting in a total skin dose of ~40 cGy over a typical treatment (20 fractions, 2 arcs/fraction, 1 cGy/arc).^12^, ^13^ Utilizing flattening filter free treatment beams could help to reduce treatment times and, therefore, the number of acquired images or, equivalently, the additional imaging dose associated with this triggered imaging technique. Other intrafraction motion monitoring systems include CyberKnife (Accuray, Sunnyvale, CA) and ExacTrac X‐ray 6D system (BrainLAB AG, Feldkirchen, Germany), which rely on ionizing radiation as in our approach.^14^, ^15^, ^16^, ^17^ However, these approaches have the advantage of a six degree of freedom couch, which may not be available in most clinics.

Overall, this approach carries certain advantages and disadvantages. An additional angular limitation of this approach as compared to the electromagnetic tracking approach is that, at certain angles such as 0 and 180, there are geometric limitations to evaluating motion in the anterior and posterior directions, which is commonly due to bladder and rectum motion. Furthermore, there are angles in which fiducials may overlap and limit automatic detection. If it is known for a given patient that the fiducials are in a configuration that results in certain angles being more informative, while other angles are less informative, then the appropriate choice of angular frequency can be made. The software does not allow selection of specific imaging angles, but one could change the angular frequency setting, as opposed to using temporal or MU frequency, to avoid less informative angles. In addition, it is recommended to always use three fiducials as opposed to one or two, to limit overlap complications (greater than three fiducials can be used, but the latency in software detection increases exponentially with increasing number of fiducials). When fiducials did overlap, the software labeled them as “not found,” this was visually confirmed, and treatment continued. We recognize that there are intrinsic limitations to this approach and with motion management using 2D information in general; however, this represents an active area of research and development.^18^, ^19^ Ideally, we would acquire 3D information with CT or even 4DCT throughout treatment, but this is not currently possible in real time as many projections are required to reconstruct 3D information. One option to overcome these limitations would be to use orthogonal imaging with both kV and MV imaging simultaneously as is currently under development,^18^, ^19^ but the Varian Truebeam v2.5 software package and technology does not allow this for clinical use. Another option would be to use other existing approaches such as electromagnetic tracking as previously discussed as well as direct real‐time anatomy tracking without the need for fiducials as with the ViewRay (ViewRay, Oakwood Village, OH) or the Elekta Unity (Elekta, Stockholm, Sweden). However, the focus of this work is to demonstrate the utility of a much more readily available technology that can be easily implemented on the widely used Varian TrueBeam system for clinics that do not have access to additional motion management technology.

While the number of patients included is believed to be sufficient for making our conclusions and demonstrating important considerations, when implementing this approach, there are many variables that may influence outcome, some of which include: specific patient anatomy, fiducial configuration (with respect to one another, with respect to the patients' anatomy, and with respect to imaging angle), the arbitrary nature of categorizing a spectrum of fiducial orientations as simply crumpled or linear, and image quality (noise) as a function of patient thickness. The potential interplay of multiple factors makes it difficult to attribute variability in the system to any one cause. For example, patients 5, 7, and 11 exhibited a higher rate of “not found” fiducials. A majority of these 'not found' data points fell at imaging angles traversing the attenuating hips, which may suggest that the fiducials may be obscured by a potential decrease in image quality; however, this was not found to be a consistent issue across all patients.

Periodic kV imaging during VMAT prostate treatments is a commercially available method to account for intrafraction motion. Appropriate use requires attention to software settings and fiducial type/configuration as well as user verification of software analysis. The “GoldSeed_1_3x5_0” setting was initially selected to best represent crumpled gold fiducial markers. However, it was found that the “Clip_1_5x4_0” actually performed better for such fiducial markers. For our clinic, the crumpled fiducial markers combined with the “Clip_1_5x4_0” software setting provided the best precision and accuracy on both phantom and patient studies. We used the Gold Anchor fiducial markers in this feasibility study because these markers were our standard, as they can be deployed with a smaller 22G preloaded needle compared to other markers. The ability to deploy a crumpled marker has also been suggested to improve localization stability due to irregular folding within tissue. Performance of this software with appropriate parameter settings may be different with alternative (e.g., cylindrical) markers. While a 10 s time interval between each kV image was used in this work, this time interval may be reduced for more precise, high‐dose treatments. However, decreasing the time interval between each kV image will increase the influence of software latencies. While these latencies are not explicitly quantified by the vendor (they are a complex function of fiducial type, orientation, configuration, and number, with computation times increasing exponentially with increasing number of fiducials), they are certainly non‐zero. In addition to software latencies, there are human latencies that factor into the visual assessment by treating therapists. This is challenging to quantify but judgments were consistently made for each image prior to the subsequent image, implying that a determination could be made in <10 s. During both latency periods, the treatment beam will continue to deliver radiation to the patient, arguing for ~3 markers (providing adequate 3D information while enabling timely results from the algorithm). Our clinic currently uses placement of three crumpled gold fiducial markers, the “Clip_1_5x4_0” setting, a 10 s interval between kV images, and a 3 mm tolerance threshold. The full functionality of the 6 degree of freedom couch was not used in this work (only translational shifts were implemented), but this would potentially allow for more accurate correction of combined shifts/rotations and might decrease the number of false‐positives and/or multi‐shift fractions. We did not evaluate the automatic beam hold feature in this software package, in which the radiation beam is gated based on automatic fiducial detection; appropriate fiducial configuration and software settings are expected to be of even greater importance in this context. The number of detected shifts was found to be surprisingly high compared to the number of shifts found to actually be required, which suggests that the algorithm is conservatively overcompensating and therefore still realistically requires human supervision. Nonetheless, by using the proper fiducial marker configuration and software settings to account for intrafraction motion, clinics may consider reduced setup margins,^20^ which may be especially valuable for hypofractionated treatments, including stereotactic body radiotherapy, where enhanced precision is required.^21^ Future work may include comparison of shift detection with periodic kV imaging and automatic marker detection (as in this work) versus with an electromagnetic tracking system.

## Conflicts of Interest

No actual or potential conflicts of interest exist.

## References

[1] Gill S , Thomas J , Fox C , et al. Acute toxicity in prostate cancer patients treated with and without image‐guided radiotherapy. Radiat Oncol. 2011;6(1):145. 22035354 10.1186/1748-717X-6-145 PMC3217047

[2] Sandler HM , Liu P‐Y , Dunn RL , et al. Reduction in patient‐reported acute morbidity in prostate cancer patients treated with 81‐Gy Intensity‐modulated radiotherapy using reduced planning target volume margins and electromagnetic tracking: assessing the impact of margin reduction study. Urology. 2010;75(5):1004–1008. 20153881 10.1016/j.urology.2009.10.072 PMC6089222

[3] Zelefsky MJ , Kollmeier M , Cox B , et al. Improved clinical outcomes with high‐dose image guided radiotherapy compared with non‐IGRT for the treatment of clinically localized prostate cancer. Int J Radiat Oncol Biol Phys. 2012;84(1):125–129. 22330997 10.1016/j.ijrobp.2011.11.047

[4] de Crevoisier R , Bayar MA , Pommier P , et al. Daily versus weekly prostate cancer image guided radiation therapy: phase 3 multicenter randomized trial. Int J Radiat Oncol Biol Phys. 2018;102(5):1420–1429. 30071296 10.1016/j.ijrobp.2018.07.2006

[5] Singh J , Greer PB , White MA , et al. Treatment‐related morbidity in prostate cancer: a comparison of 3‐dimensional conformal radiation therapy with and without image guidance using implanted fiducial markers. Int J Radiat Oncol Biol Phys. 2013;85(4):1018–1023. 23040222 10.1016/j.ijrobp.2012.07.2376

[6] Wortel RC , Incrocci L , Pos FJ , et al. Acute toxicity after image‐guided intensity modulated radiation therapy compared to 3D conformal radiation therapy in prostate cancer patients. Int J Radiat Oncol Biol Phys. 2015;91(4):737–744. 25752386 10.1016/j.ijrobp.2014.12.017

[7] Chaurasia AR , Sun KJ , Premo C , et al. Evaluating the potential benefit of reduced planning target volume margins for low and intermediate risk patients with prostate cancer using real‐time electromagnetic tracking. Adv Radiat Oncol. 2018;3(4):630–638. 30370364 10.1016/j.adro.2018.06.004 PMC6200876

[8] Vanhanen A , Syrén H , Kapanen M . Localization accuracy of two electromagnetic tracking systems in prostate cancer radiotherapy: a comparison with fiducial marker based kilovoltage imaging. Phys Med. 2018;56:10–18. 30527084 10.1016/j.ejmp.2018.11.007

[9] Takakura T , Mizowaki T , Nakata M , et al. The geometric accuracy of frameless stereotactic radiosurgery using a 6D robotic couch system. Phys Med Biol. 2010;55(1):1–10. 19949261 10.1088/0031-9155/55/1/001

[10] Hamilton DG , McKenzie DP , Perkins AE . Comparison between electromagnetic transponders and radiographic imaging for prostate localization: A pelvic phantom study with rotations and translations. J Appl Clin Med Phys. 2017;18(5):43–53. 28699243 10.1002/acm2.12119 PMC5875817

[11] Langen KM , Willoughby TR , Meeks SL , et al. Observations on real‐time prostate gland motion using electromagnetic tracking. Int J Radiat Oncol. 2008;71(4):1084–1090. 10.1016/j.ijrobp.2007.11.054 18280057

[12] Murphy MJ , Balter J , Balter S , et al. The management of imaging dose during image‐guided radiotherapy: report of the AAPM Task Group 75. Med Phys. 2007;34(10):4041–4063. 17985650 10.1118/1.2775667

[13] Ding GX , Munro P . Radiation exposure to patients from image guidance procedures and techniques to reduce the imaging dose. Radiother Oncol. 2013;108(1):91–98. 23830468 10.1016/j.radonc.2013.05.034

[14] O'neill AGM , Jain S , Hounsell AR , O'sullivan JM . Fiducial marker guided prostate radiotherapy: a review. Br J Radiol. 2016;89(1068):20160296. 27585736 10.1259/bjr.20160296 PMC5604907

[15] Xie Y , Djajaputra D , King CR , Hossain S , Ma L , Xing L . Intrafractional motion of the prostate during hypofractionated radiotherapy. Int J Radiat Oncol Biol Phys. 2008;72(1):236–246. 18722274 10.1016/j.ijrobp.2008.04.051 PMC2725181

[16] Shi C , Tazi A , Fang DX , Iannuzzi C . Study of ExacTrac X‐ray 6D IGRT setup uncertainty for marker‐based prostate IMRT treatment. J Appl Clin Med Phys. 2012;13(3):35–42. 10.1120/jacmp.v13i3.3757 PMC5716561 22584176

[17] Holmes OE , Gratton J , Szanto J , et al. Reducing errors in prostate tracking with an improved fiducial implantation protocol for CyberKnife based stereotactic body radiotherapy (SBRT). J Radiosurgery SBRT. 2018;5(3):217–227. Accessed August 18, 2019. PMC6018049 29988326

[18] Ren L , Zhang Y , Yin FF . A limited‐angle intrafraction verification (LIVE) system for radiation therapy. Med Phys. 2014;41(2):020701. 24506590 10.1118/1.4861820

[19] Zhang Y , Deng X , Yin F‐F , Ren L . Image acquisition optimization of a limited‐angle intrafraction verification (LIVE) system for lung radiotherapy. Med Phys. 2018;45(1):340–351. 29091287 10.1002/mp.12647 PMC5774243

[20] Rosario T , van der Weide L , Admiraal M , Piet M , Slotman B , Cuijpers J . Toward planning target volume margin reduction for the prostate using intrafraction motion correction with online kV imaging and automatic detection of implanted gold seeds. Pract Radiat Oncol. 2018;8(6):422–428. 29907506 10.1016/j.prro.2018.04.008

[21] Carrasquilla M , Jones K , Sen N , et al. Quantification of prostate motion during stereotactic body radiation therapy boost using on‐demand imaging and fiducial markers: preliminary results from a phase 2 study. Int J Radiat Oncol Biol Phys. 2017;99(2):E218.

